# Loss of Skeletal Muscle HIF-1α Results in Altered Exercise Endurance

**DOI:** 10.1371/journal.pbio.0020288

**Published:** 2004-08-24

**Authors:** Steven D Mason, Richard A Howlett, Matthew J Kim, I. Mark Olfert, Michael C Hogan, Wayne McNulty, Reed P Hickey, Peter D Wagner, C. Ronald Kahn, Frank J Giordano, Randall S Johnson

**Affiliations:** **1**Molecular Biology Section, Division of BiologySchool of Medicine, University of California, San Diego, CaliforniaUnited States of America; **2**Division of Physiology, School of MedicineUniversity of California, San Diego, CaliforniaUnited States of America; **3**Cardiology Division, Yale University Medical SchoolNew Haven, Connecticut United States of America; **4**Joslin Diabetes Foundation, Harvard Medical SchoolBoston, MassachusettsUnited States of America

## Abstract

The physiological flux of oxygen is extreme in exercising skeletal muscle. Hypoxia is thus a critical parameter in muscle function, influencing production of ATP, utilization of energy-producing substrates, and manufacture of exhaustion-inducing metabolites. Glycolysis is the central source of anaerobic energy in animals, and this metabolic pathway is regulated under low-oxygen conditions by the transcription factor hypoxia-inducible factor 1α (HIF-1α). To determine the role of HIF-1α in regulating skeletal muscle function, we tissue-specifically deleted the gene encoding the factor in skeletal muscle. Significant exercise-induced changes in expression of genes are decreased or absent in the skeletal-muscle HIF-1α knockout mice (HIF-1α KOs); changes in activities of glycolytic enzymes are seen as well. There is an increase in activity of rate-limiting enzymes of the mitochondria in the muscles of HIF-1α KOs, indicating that the citric acid cycle and increased fatty acid oxidation may be compensating for decreased flow through the glycolytic pathway. This is corroborated by a finding of no significant decreases in muscle ATP, but significantly decreased amounts of lactate in the serum of exercising HIF-1α KOs. This metabolic shift away from glycolysis and toward oxidation has the consequence of increasing exercise times in the HIF-1α KOs. However, repeated exercise trials give rise to extensive muscle damage in HIF-1α KOs, ultimately resulting in greatly reduced exercise times relative to wild-type animals. The muscle damage seen is similar to that detected in humans in diseases caused by deficiencies in skeletal muscle glycogenolysis and glycolysis. Thus, these results demonstrate an important role for the HIF-1 pathway in the metabolic control of muscle function.

## Abstract 2

Mice that can't express HIF-1α in skeletal muscle don't induce glycolytic genes and thus switch to aerobic metabolism allowing them to run uphill longer than the their wild-type littermates

## Introduction

During exercise in normoxia, the partial pressure of oxygen in muscle tissue has been shown to dip to as low as 3.1 mm Hg, whereas in the capillary, it remains at 38 mm Hg (Hoppeler et al. 2003). In order to maintain effort, skeletal muscle exertion must be able to rely on pathways designed to help the tissue cope with oxygen stress after oxygen delivery capacity is exceeded. A switch between aerobic and nonaerobic metabolism during strenuous exertion requires mechanisms to adjust metabolic function, and this need is acute in extended exertion in skeletal muscle. It is clear that the transcription factor hypoxia-inducible factor 1α (HIF-1α) is an essential factor in maintenance of ATP levels in cells (Seagroves et al. 2001). In fact, although HIF-1α is typically thought of as acting only during hypoxia, its loss has an effect on both normoxic and hypoxic ATP levels in a number of tissue types (Seagroves et al. 2001; Cramer et al. 2003), and this implicates the factor in regulation of metabolic function even during conditions of normal physiologic oxygenation.

In skeletal muscle, signaling of fatigue has been studied extensively, and signaling of exhaustion involves, to some degree, elevated systemic lactic acid, a by-product of the glycolytic pathway of metabolism (Myers and Ashley 1997). Thus, the glycolytic pathway is intrinsically involved in muscle function and fatigue, and this in turn is linked to the response to hypoxia. To understand how the primary hypoxia-responsive transcription factor controls skeletal muscle function, we targeted mouse skeletal muscle for tissue-specific deletion of *HIF-1α* via the use of a conditionally targeted allele of the gene (Ryan et al. 2000; Schipani et al. 2001). This mouse strain was crossed into a strain transgenic for the skeletal-muscle-specific muscle creatine kinase (MCK) promoter, which drives expression of the *cre* recombinase gene (Bruning et al. 1998; Sauer 1998). We found that loss of the regulation of hypoxic response in muscle has a profound effect on the function of the muscle during exertion, with effects that mimic human metabolic myopathies.

## Results/Discussion

**Figure 1:**
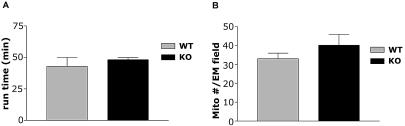
Exercise Capacity of Cardiac HIF-1α KOs and HIF-1α/MCK/c*re* Mitochondrial Density (A) Mice lacking cardiac HIF-1α perform no differently in endurance running trials than WT mice, showing that the increase in exercise capacity seen in MCK/Cre mice is due to deletion of HIF-1α in skeletal muscle, not cardiac tissue. (B) Mice lacking skeletal muscle HIF-1α have a slight but nonsignificant increase in mitochondrial density as measured by the number of mitochondria per electron microscope field of view.

**Figure 2:**
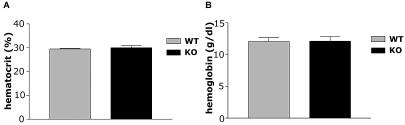
Hematocrit and Hemoglobin Levels in HIF-1α KOs and WT Mice (A) Hematocrit levels are virtually identical in both HIF-1α KOs (*n* = 3) and WT (*n* = 4) mice, indicating that loss of HIF-1α in skeletal muscle does not affect oxygen carrying capacity of the blood. (B) In addition to similar hematocrit levels, WT mice and HIF-1α KOs have very close blood hemoglobin levels.

In 4-mo–old mice with the skeletal-muscle *HIF-1α* gene knocked out (HIF-1α KOs), the frequency of excision was evaluated through real-time PCR techniques. We saw deletion frequencies consistent with those described previously for this *cre* recombinase transgene (Bruning et al. 1998) with some variation in penetration; mean frequency of deletion was 54.9%, with the highest frequency of muscle-specific deletion of HIF-1α being 72% in the gastrocnemius of 4-mo–old mice homozygous for the *loxP*-flanked allele (Table 1). This transgene is expressed at a lower level in cardiac tissue, and cardiac deletion was detected (Table 1); however, none of the phenotypes described below were seen in cardiac myocyte-specific deletions of HIF-1α (Figure 1A). Gross muscle sections were evaluated histologically to evaluate both vascularization and fiber type (Tables 2 and 3), and ultrastructurally to determine number of mitochondria (Figure 1B). No changes were detected in any of these features in HIF-1α KOs, except for a slight but statistically significant decrease in type IIA fibers in the soleus muscles (Table 3). Similar hematocrit and blood hemoglobin levels were seen in HIF-1α KOs and wild-type (WT) mice (Figure 2).

**Table 1:**
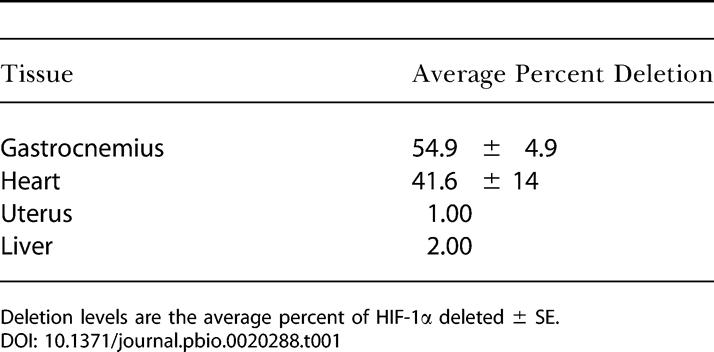
Excision of HIF-1α in Various Tissues

Deletion levels are the average percent of HIF-1α deleted ± SE

**Table 2:**
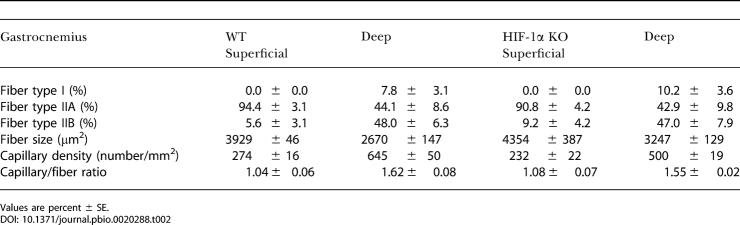
Fiber Typing of Gastrocnemius Muscle

Values are percent ± SE

**Table 3:**
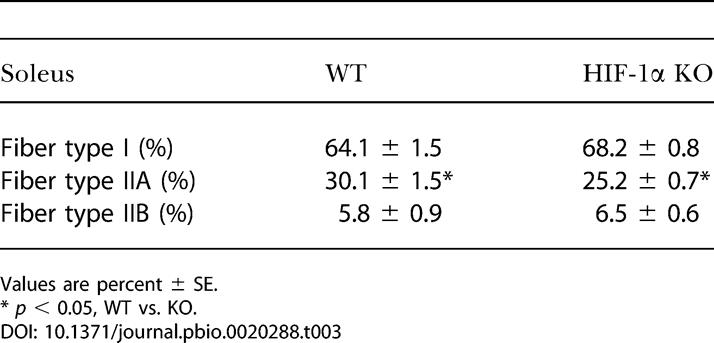
Fiber Typing of Soleus Muscle

Values are percent ± SE

* *p* < 0.05, WT vs. KO

As can be seen in Table 4, significant changes in HIF-1α–dependent gene expression occur in muscle during exercise, including changes in genes involved in glucose transport and metabolism. Vascular endothelial growth factor (VEGF), which increases vascular permeability, and glucose transporter 4 (GLUT4), the muscle-specific glucose transporter, show increased levels in exercise and likely increase the availability of glucose to the muscle. The muscle-specific form of phosphofructokinase (PFK-M), phosphoglycerate kinase (PGK), and lactate dehydrogenase-A (LDH-A) are also up-regulated at the mRNA level by exercise, and this up-regulation is inhibited by the loss of HIF-1α, further demonstrating that HIF-1α is important for transcriptional response during skeletal muscle activity.

**Table 4:**
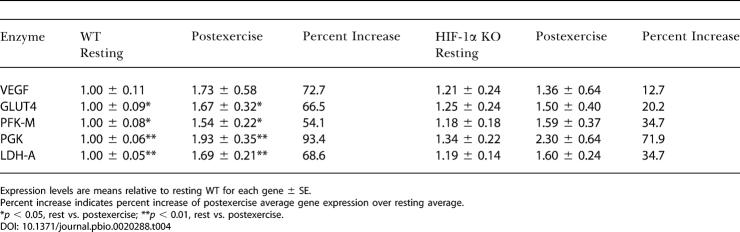
Relative Gene Expression Levels

Expression levels are means relative to resting WT for each gene ± SE

Percent increase indicates percent increase of postexercise average gene expression over resting average

*p < 0.05, rest vs. postexercise; **p < 0.01, rest vs. postexercise

**Figure 3:**
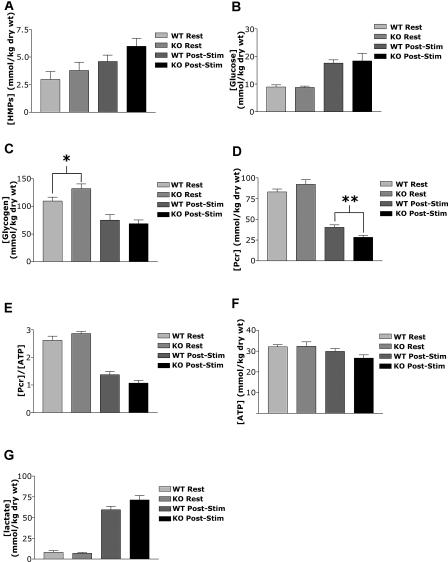
Intramuscular Metabolite Levels at Rest and Following Stimulation (A) Glycolytic intermediates were measured from gastrocnemius muscles following the isolated stimulation protocol. Resting values represent levels in the unstimulated gastrocnemius from the same animals. HIF-1α KOs had a trend toward greater accumulated levels of HMPs during the stimulation protocol, although the difference did not reach statistical significance (*p* = 0.10). This difference could be indicative of a blockage in the glycolytic pathway at PFK. (B) No significant differences were seen between HIF-1α KOs and WT intramuscular glucose levels at rest or following stimulation. Both HIF-1α KO and WT muscles were able to significantly increase glucose uptake, leading to greater levels of intramuscular glucose in response to stimulation (WT, *p* < 0.001; KO, *p* < 0.05). (C) HIF-1α KOs have more stored glycogen than do WT mice. Glycogen levels were measured following the same stimulation protocol as in (B). The change in glycogen from rest to poststimulation was also greater in the HIF-1α KOs, indicating that they metabolized more glycogen in response to stimulation (*p* < 0.01; *p < 0.05, WT at rest vs. KO at rest). (D) HIF-1α KOs utilize more PCr in response to stimulation than do WT mice. Similar levels of PCr were seen at rest, but HIF-1α KOs metabolized significantly more during stimulation (*p* < 0.05) and had much lower levels following the protocol (**p < 0.01, WT poststimulation vs. KO poststimulation). (E) A trend toward lower PCr/ATP concentration ratios was seen in HIF-1α KOs relative to WT mice following stimulation, although the difference did not quite reach statistical significance (*p* < 0.10). A trend toward a greater drop from rest to poststimulation in the PCr/ATP ratio was also seen in HIF-1α KOs following stimulation (*p* < 0.10), indicating that they had to rely more heavily on PCr for ATP generation. (F) Slight but nonsignificant differences were seen in whole-muscle ATP levels at rest or following stimulation. Although HIF-1α KOs exhibited altered substrate utilization, they were able to meet their ATP demands during the protocol. (G) Both HIF-1α KOs and WT animals produced significant intramuscular lactate during the stimulation protocol; however, there was no significant difference in the amount produced by either genotype. Resting intramuscular lactate levels were also similar for WTs and HIF-1α KOs.

**Figure 4:**
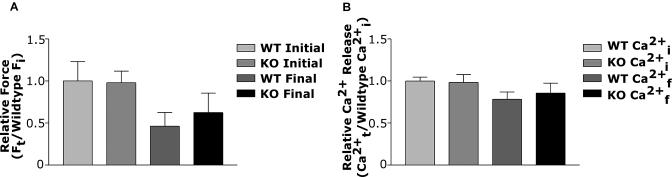
Force Generation and Ca^2+^ Release in Isolated Muscle Fibers during Stimulation (A) No differences were seen in total force generation in isolated muscle fibers. Mechanically dissected fibers from the flexor brevis muscle were subjected to a fatiguing protocol. Neither initial nor final forces differed between HIF-1α KO and WT fibers. (B) Ca^2+^ release and reuptake in HIF-1α KO and WT fibers was not different during the stimulation protocol. Ca^2+^ levels were measured in individual fibers through use of fura-2 Ca^2+^ indicator. The altered substrate utilization did not affect the ability of the fibers to maintain proper Ca^2+^ flux.

In Table 5, we show the changes in enzymatic activity in a number of key glycolytic enzymes affected by deletion of HIF-1α. As can be seen from the data, several of the enzymes assayed showed a decrease in activity in response to exercise. In particular, the activity of one of the key rate-limiting enzymes, PFK, was significantly lower following exercise in HIF-1α KOs compared to WT mice, indicating that HIF-1α KOs may have difficulty maintaining optimal PFK activity. The responses of other glycolytic enzymes to exercise were fairly similar between WT mice and HIF-1α KOs. These include no significant changes in phosphoglucose isomerase activity and significant, yet similar, decreases in aldolase, glyceraldehyde 3-phosphate dehydrogenase, and PGK activities. An exception to this is that WT muscles were able to significantly increase pyruvate kinase (PK) activity (see Table 4; *p* < 0.05). LDH activity was also increased in the WT mice, although the level did not reach statistical significance. Activities of both PK and LDH were not significantly changed in HIF-1α KO muscles following exercise. Increased activities of PK, and subsequently LDH, could be expected to lead to increased levels of lactate in the WT mice relative to HIF-1α KOs.In Figure 3A, it can be seen that the decrease in PFK activity in the HIF-1α KOs is correlated with a trend approaching significance (*p* = 0.10) toward an increased amount of hexose monophosphates (HMPs), which are pre-PFK glycolytic metabolites, following stimulation of the HIF-1α KO muscle. This increase was not due to differences in glucose uptake, since animals of both genotypes were able to significantly increase intramuscular glucose to a similar degree (Figure 3B). Consistent with decreased flow through the glycolytic pathway, however, the increased amount of HMPs was correlated with increased muscle glycogenolysis (Figure 3C) and increased depletion of phosphocreatine (PCr) (Figure 3D), with a resultant decrease in the PCr/ATP ratio in HIF-1α KO muscle (Figure 3E), although there was only a nonsignificant drop in overall muscle ATP concentrations (Figure 3F). Intramuscular levels of lactate did increase in both HIF-1α KOs and WT mice during stimulation, although lactate accumulation did not differ significantly between them (Figure 3G). In order to evaluate whether these changes had any effect on overall muscle force, we measured force and calcium release in isolated single fibers; as can be seen in Figure 4A and 4B, there were no significant changes in these parameters, indicating that the muscle can compensate at this level for the metabolic changes induced by loss of HIF-1α.

**Table 5:**
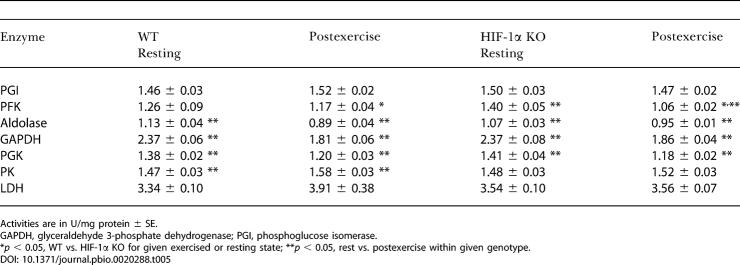
Glycolytic Enzyme Activity Levels from Gastrocnemius Muscles

Activities are in U/mg protein ± SE

GAPDH, glyceraldehyde 3-phosphate dehydrogenase; PGI, phosphoglucose isomerase

*p < 0.05, WT vs. HIF-1α KO for given exercised or resting state; **p < 0.05, rest vs. postexercise within given genotype

**Figure 5:**
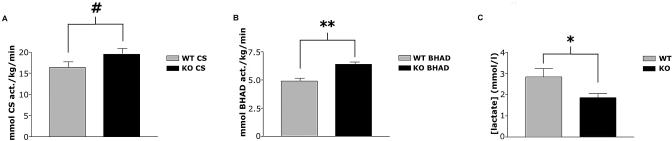
Oxidative Metabolism and Serum Lactate Production in HIF-1α KOs and WT Mice (A) HIF-1α KOs have higher resting levels of CS activity. CS is an enzyme in the Krebs cycle that can be regulated allosterically by ATP levels. Increased CS activity is indicative of increased muscle oxidative capacity, which is common in patients with glycogenolytic or glycolytic myopathies (^#^ *p* < 0.10, KO vs. WT). (B) HIF-1α KOs have higher resting levels of B-HAD activity, which is indicative of a greater ability to oxidize fatty acids (**p < 0.01, WT vs. KO). (C) Lower serum lactate levels were seen in HIF-1α KOs following a timed 25-minute run (*p < 0.05, WT vs. KO).

Given altered levels of glycolytic throughput without significant changes in intramuscular ATP levels, it is likely that there is increased activity of oxidative pathways in the HIF-1α KO muscle. Increased muscle oxidative activity is typical in patients with myopathies involving muscle glycolysis or glycogenolysis, including phosphofructokinase disease (PFKD) and McArdle's disease (Vissing et al. 1996). We analyzed the activity of citrate synthase (CS), a key allosteric enzyme of the citric acid cycle, in WT and HIF-1α KO muscle (Figure 5A), and found that it was up-regulated in HIF-1α KOs. CS is a mitochondrial enzyme that responds to decreases in ATP concentration allosterically, allowing for increased oxidative activity in the mitochondria. In addition, significant up-regulation of the mitochondrial enzyme beta-hydroxyacyl CoA dehydrogenase (B-HAD) was seen in HIF-1α KO muscle (Figure 5B). B-HAD is also affected by energy levels in the cell, and decreases in NADH/NAD^+^ concentration ratios cause the enzyme to increase mitochondrial oxidation of fatty acids (Nelson and Cox 2000). Increased activity of oxidative pathways in the muscle should result in more rapid lactate clearance, as in fact occurs in PFKD patients during exercise; this phenomenon gives rise to a “second wind” in these patients, and under some circumstances allows for an increase in exercise endurance (Vissing et al. 1996; Haller and Vissing 2002), although this was disputed in one recent study (Haller and Vissing 2004). This decreased lactate accumulation postexercise clearly occurs in the HIF-1α KOs, as can be seen in Figure 5C. This systemically lower level of lactate postexercise indicates that there may be a shift toward a more oxidative metabolism in skeletal muscle.

**Figure 6:**
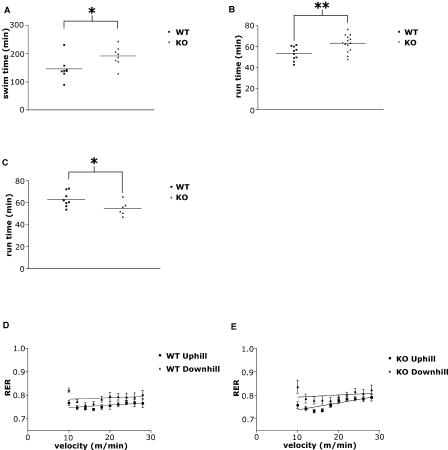
Endurance Capabilities of Untrained Mice (A) HIF-1α KOs have greater endurance in swimming tests as shown by their ability to swim on average more than 45 min longer than WT (*p < 0.05, WT vs. KO). (B) HIF-1α KOs have greater endurance than WT mice in uphill running tests. Although only a 10-min difference is seen between run times, it is to be noted that because of the protocol, this 10 min included two velocity increases (**p < 0.01, WT vs. KO). (C) HIF-1α KOs have less endurance than WT mice in downhill running tests. The same protocol was used as in Figure 4A, except the mice were run on a 10° decline (*p < 0.05, WT vs. KO). (D) RER uphill vs. downhill in WT mice. As would be expected from eccentric exercises relying more heavily on glycolytic fibers, the RER values are higher in mice running downhill than in those running uphill. (E) RER uphill vs. downhill in HIF-1α KOs. Once again, higher RER values are observed for mice running downhill than those running uphill.

As mentioned above, patients with muscle glycolytic deficiencies demonstrate both increased exercise-induced muscle damage and a “second wind”; the latter phenomenon allows them to exercise for extended periods of time at submaximal levels. This is thought to be due to an increase in rates of oxidative ATP production, and a decreased utilization of and need for muscle glycogen (Vissing et al. 1996; Haller and Vissing 2002). To assess whether this is also the case in the HIF-1α KOs, both WT mice and HIF-1α KOs were subjected to endurance tests to assess muscle function. To first determine whether HIF-1α KOs were capable of extended activity during exercise, the animals were given a swimming endurance test. As can be seen in Figure 6A, HIF-1α KOs were capable of significantly longer-duration swimming activity when compared to matched WT controls (*p* < 0.05).

Further testing was done to determine the parameters of this increased endurance. HIF-1α KOs were run on an enclosed treadmill, with a 5° incline and an initial velocity of 10 m/min, with an increase in velocity every 5 min. In their first runs, HIF-1α KOs again had significantly greater endurance, as shown by their consistently longer run times compared to WT controls (*p* < 0.01, Figure 6B).

As it has been shown that muscle groups and fibers respond differently to eccentric exercise (i.e., downhill running) than to concentric exercise (i.e., uphill running) (Nardone and Schieppati 1988), mice from both genotypes were run on a 10° decline with the same velocity and time parameters as in the uphill runs. Eccentric exercises have been shown to recruit primarily fast-twitch glycolytic fibers for contraction, as opposed to the traditional recruitment of slower, smaller, oxidative motor units in concentric contraction, where animals with an increased capacity for muscle oxidation would be at an advantage (Nardone and Schieppati 1988). Now, the trend from swimming and uphill running tests was reversed, with WT mice able to run for a significantly longer time than HIF-1α KOs (*p* < 0.05, Figure 6C). Within genotypes, WT mice ran for significantly longer times downhill than uphill (*p* < 0.01); HIF-1α KOs did the reverse, and ran for significantly shorter times downhill than uphill (*p* < 0.05). Substrate utilization confirms the shift toward glycolytic fibers in downhill running; both genotypes had higher average respiratory exchange ratio (RER) values when running downhill compared with running uphill (Figure 6D and 6E).

**Figure 7:**
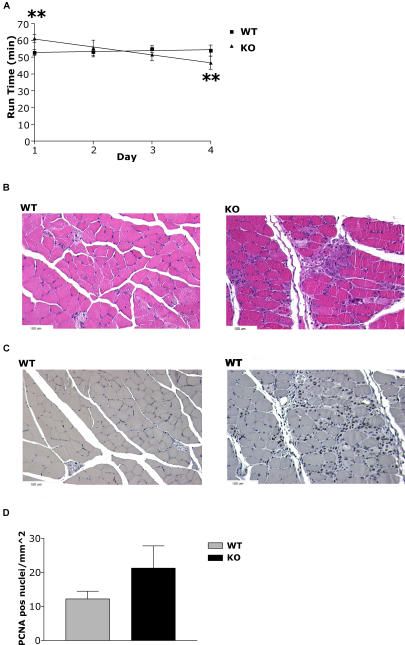
Increased Muscle Damage in HIF-1α KOs Following Repeated Exercise (A) WT mice and HIF-1α KOs underwent a 4-d endurance test, in which animals were run to exhaustion on each of four successive days with a minimum of 22 h rest between trials. HIF-1α KOs demonstrated initially greater endurance under the protocol; however, by the second day, their endurance advantage was eliminated, and by the fourth day, HIF-1α KOs were running for a significantly shorter time (**p < 0.01) than on the first day, while WT animals were running for approximately similar times as on the first day. Repeated measures ANOVA revealed that the decrease in performance on each successive day was unique to HIF-1α KOs (*p* < 0.05). (B) Example of hematoxylin and eosin staining of gastrocnemius muscles after 1 d of recovery by mice after the 4-d endurance test. Evidence of greater damage can be seen in HIF-1α KO muscles compared to WT muscles. (C) Example of PCNA staining of gastrocnemius muscles from exercised mice, demonstrating increased levels of muscle regeneration in HIF-1α KOs. (D) Number of PCNA-positive nuclei per square millimeter in gastrocnemius muscles of WT mice (*n* = 5) and HIF-1α KOs (*n* = 7) that ran repeatedly for 4 d. Although HIF-1α KOs have almost twice as many PCNA-positive nuclei per square millimeter, the difference is not significant, because of wild variations in that population. F-test analysis of the data reveals that the variance is much greater in the HIF-1α KO population than the WT population (*p* < 0.05).

PFKD and McArdle's disease demonstrate significant myopathic effects in muscle, including soreness and cramping induced by bouts of exercise. After 1 d of recovery from endurance testing, HIF-1α KOs had increased levels of the MM isoform of creatine kinase in their serum (unpublished data), indicative of skeletal muscle damage. To further investigate this finding, mice were run on a treadmill daily for 4 d. By the second day, the trend for increased endurance in the HIF-1α KOs was absent, and by the final day, HIF-1α KOs were running for significantly shorter times than they had on the first day (*p* < 0.01, Figure 7A). In addition, a repeated measures ANOVA performed on run times showed that the response of the HIF-1α KOs to the protocol was significantly different than that of the WT mice (*p* < 0.05). Histological examination of gastrocnemius tissue following 1 d of recovery revealed significantly greater amounts of muscle damage in HIF-1α KO tissue than WT tissue (Figure 7B). Staining of the tissue for proliferating cellular nuclear antigen (PCNA) and counts of positive nuclei (Olive et al. 1995) also revealed more cell division in HIF-1α KOs than in WTs, another indication that HIF-1α KOs had been subject to greater tissue damage (Figure 7C and 7D).

**Figure 8:**
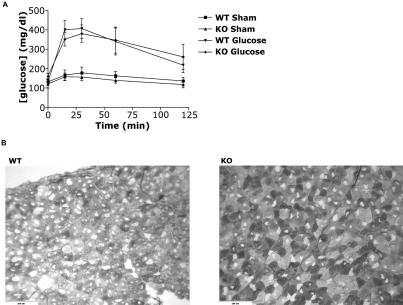
Glucose Tolerance and Glycogen Storage (A) No significant differences were seen in resting blood glucose levels in HIF-1α KOs or WT mice. Following injection of glucose at a dosage of 2 g/kg, no differences were seen in the maximum levels of blood glucose or the rate of glucose disappearance in either genotype. (B) Representative PAS staining of gastrocnemius muscle from WT mice and HIF-1α KOs. HIF-1α KOs demonstrate darker staining, indicating more stored glycogen.

As noted above, both PFKD and McArdle's disease are marked by increased resting intramuscular levels of glycogen, a failure of serum lactate to rise during exertion, an exercise-induced “second wind,” and signs of muscle damage following exertion, including elevated levels of creatine kinase in the serum (Tarui et al. 1965; Layzer et al. 1967). In addition, PFKD is characterized by elevated levels of HMPs (Tarui et al. 1965; Layzer et al. 1967; Argov et al. 1987; Grehl et al. 1998) and greater PCr utilization during contraction (Argov et al. 1987; Grehl et al. 1998). We see many of these hallmarks of muscle deficiencies in glycolytic processing in HIF-1α KOs. The effects are not likely due to glucose uptake, as WT and HIF-1α KO intramuscular glucose levels were not different at rest or following stimulation (see Figure 3B), and both types of mice responded similarly to a glucose tolerance test (Figure 8A). Periodic acid–Schiff (PAS) staining of tissue from mice of both genotypes gave further demonstration of increased glycogen levels in resting muscles from HIF-1α KOs (Figure 8B).

Given the differences in performance observed in the HIF-1α KOs in eccentric and concentric exercise, it is clear that the HIF-1 pathway and hypoxic response have a central role in determining the capacity for work and endurance through regulation of glycolysis. It is also clear that these mice will provide an important model system to investigate the physiology of muscle response during work and oxygen depletion, and may be useful as a model for a group of very debilitating myopathic syndromes in humans.

## Materials and Methods

### 

#### Mouse strains and crosses.

Mice were generated from *HIF-1α loxP*-flanked allele mouse stocks backcrossed into a C57Bl6/J background. These were crossed into a C57Bl6/J strain containing the *MCK/cre* transgene. Controls were in all cases littermates that were genotyped as containing only the *loxP*-flanked *HIF-1α* allele or only the *MCK/cre* transgene. No phenotypic differences were seen in the two controls, so they were considered interchangeably as WT control animals.

#### Genotyping and real-time PCR for HIF-1α deletion

Mice from the above crosses were genotyped using DNA extracted from tail sections. DNA was then extracted from the gastrocnemius, heart, liver, and uterus of eight 4-mo–old, *loxP*-flanked *HIF-1α–*positive and *MCK/cre*–positive mice. *HIF-1α* levels were measured by real-time PCR analysis using the Universal PCR Master Mix Kit (Applied Biosystems, Foster City, California, United States) and the ABI Prism 7700 Sequence Detector (Applied Biosystems). Conditions for the PCR were one 10-min incubation at 95 °C (polymerase activation), followed by 40 cycles of 15 s at 95 °C (denaturation) and 1 min at 60 °C (anneal/extend). The degree of excision was calculated by comparing *HIF-1α* DNA levels to *c-Jun* DNA levels. *HIF-1α* real-time PCR primers and probe were as follows: forward primer, HIFLOX501/F 5′-CTATGGAGGCCAGAAGAGGGTAT-3′; reverse primer, HIFLOX574/R 5′-CCCACATCAGGTGGCTCATAA-3′; probe, HIFLOX/P 5′-(6FAM)AGATCCCTTGAAGCTAG(MGBNFQ)-3′.

#### Muscle histology and electron microscopy.

Paraffined gastrocnemius sections were deparaffinized and stained with Gill II hematoxylin. Sections were then washed successively in water, a bluing agent, water again, and 95% ethanol, and restained with eosin. Hematoxylin and eosin staining was performed by the University of California at San Diego (UCSD) Cancer Center Histology Resource (La Jolla, California, United States). Imaging was performed on sections mounted on slides using Cytoseal 60 (VWR, West Chester, Pennsylvania, United States). Electron microscopy was performed by standard methods on gastrocnemius muscle. Briefly, fixation was by 25.5% glutaraldehyde in 0.1 M sodium cacodylate buffer (pH 7.4). Postfix was in 1% osmium tetroxide. The section was stained in 2% uranyl acetate in sodium maleate buffer (pH 5.2), then placed in Epon resin (VWR, West Chester, Pennsylvania, United States), and cured overnight at 60 °C. Fiber typing was performed using the metachromatic dye ATPase method (Ogilvie and Feeback 1990). PAS staining was performed as has been described (Bancroft and Stevens 1996).

#### Assessment of exhaustion.

Untrained, age-matched WT mice and HIF-1α KOs (WT, *n* = 10; KO, *n* = 14) were run either on an Omnipacer treadmill (Columbus Instruments, Columbus, Ohio, United States) or on an enclosed-chamber modular treadmill (Columbus Instruments) with a 5° incline at an initial velocity of 10 m/min. Velocity was increased by 2 m/min every 5 min during the assessment. Exhaustion was determined to be the point at which the animal would not resume running when provoked through a low-voltage power grid. Gas flow (O_2_ and CO_2_) into and out of the enclosed chamber treadmill was monitored using the Paramax O_2_ sensor and a CO_2_ sensor (Columbus Instruments) and analyzed using Oxymax software (Columbus Instruments) to determine metabolic parameters. The downhill running assessment (WT, *n* = 8; KO, *n* = 6) was carried out in the enclosed-chamber modular treadmill at a 10° decline using the same protocol as above.

In the swimming exhaustion assessment, a second group of WT and HIF-1α KOs (*n* = 8 for each class) was placed in a 30 °C water bath with mild turbulence. Exhaustion was determined to be the point at which the animal experienced three successive periods below the surface of more than 3 s.

#### Isolated stimulation and metabolic analysis.

The Achilles tendon was surgically freed from live, anesthetized mice (WT, *n* = 8; KO, *n* = 6) and attached to a force transducer to record contractile force. Muscles were electrically stimulated through excitation of the sciatic nerve. Stimulation was in the form of 8–10-V direct titanic contractions using 200-ms trains at 70 Hz with 0.2 ms duration. Initial frequency of tetanic contraction was one every 8 s and was increased every 2 minutes to one every 4 s and one every 3 s, up to the end point of 6 min. Isolated muscles were then immediately harvested and snap-frozen for ATP, lactate, phosphocreatine, and glycogen analyses. Samples were freeze-dried and analyzed by enzymatic assay as has been previously described (Bergmeyer 1974). The unstimulated gastrocnemius muscle from each mouse was used as a resting control.

#### Real-time PCR measurement of gene expression.

For basal gene expression levels, total RNA was isolated from gastrocnemius tissue from seven WT and five HIF-1α KOs using RNA-Bee (Tel-Test, Friendswood, Texas, United States). Reverse transcription was performed using the Superscript First Stand Synthesis System for RT-PCR (Invitrogen, Carlsbad, California, United States). Amplification was performed using the ABIPrism 7700 as described above. Reverse transcription real-time PCR primers and probes were as follows. For PGK-1: reverse primer, PGK/R 5′-CAGGACCATTCCAAACAATCTG-3′; forward primer, PGK/F 5′-CTGTGGTACTGAGAGCAGCAAGA-3′; probe, PGK/P 5′-(6∼FAM)TAGCTCGACCCA-CAGCCTCGGCATAT(TAMRA)-(phosphate)-3′. For VEGF-A: reverse primer, VEGF/R 5′-ATCCGCATGATCTGCATGG-3′; forward primer, VEGF/F 5′-AGTCCCATGAAGTGATCAAGTTCA-3; probe, VEGF/P (6∼FAM)TGCCCACGTCAGAGAGCAACATCAC(BHQ∼6∼FAM). For GLUT4: reverse primer, GLUT-4/R 5′-CCCATGCCGACAATGAAGTT-3′; forward primer, GLUT-4/F 5′-TGTGGCCTTCTTTGAGATTGG-3′; probe, GLUT-4/P 5′(6-FAM)TGGCCCCATTCCCTGGTTCATT(BHQ1-Q)-3′. For PFK-M: reverse primer, PFK-M/R 5′-AAGTCGTGCAGATGGTGTTCAG-3′; forward primer, PFK-M/F 5′-GCCACGGTTTCCAATAACGT-3′; probe, PFK-M/P 5′-(6-FAM)CCTGGGTCAGACTTCAGCATCGGG(BHQ1-Q)-3′. For LDH-A: reverse primer, LDH-A/R 5′-ATGCACCCGCCTAAGGTTCTT-3′; forward primer, LDH-A/F 5′-TGCCTACGAGGTGATCAAGCT-3′; probe, LDH-A/P 5′-(6- FAM)TGGCAGACTTGGCTGAGAGCAT(BHQ1-Q)-3′.

For changes in gene expression due to exercise, age-matched male mice (WT, *n* = 5; KO, *n* = 6) were run on a treadmill at 25 m/min for 30 min. Following the run, mice were euthanized and RNA was isolated and analyzed as described above.

#### Analysis of enzyme activity levels

For changes in enzyme activity levels with exercise, mice (WT, *n* = 5; KO, *n* = 12) were run on a treadmill using the same protocol as for the gene expression analysis. Tissue was harvested after the run and from resting mice (WT, *n* = 6; KO, *n* = 10), and enzymes were extracted and analyzed spectrophotometrically as has been described (Reichmann et al. 1983), with the exception that fructose 1,6-bisphosphate was replaced with fructose 2,6-bisphosphate for stabilization of PFK. Units of activity were normalized to milligrams of total protein using a BCA protein quantification kit (Pierce Biotechnology, Rockford, Illinois, United States).

#### Creatine kinase, serum lactate, hematocrit, and hemoglobin levels.

Creatine kinase levels were analyzed from serum from WT mice and HIF-1α KOs 24 h after running-induced exhaustion using a kit from Sigma (St. Louis, Missouri, United States). Creatine kinase isoforms were analyzed enzymatically and then fractionated by gel electrophoresis. Serum lactate levels were analyzed by the UCSD Comparative Neuromuscular Laboratory from blood obtained by cardiac puncture from six WT mice and six HIF-1α KOs following 25 min running time on the treadmill ramp at 25 m/min. Hematocrit and hemoglobin levels were measured from resting mice (WT, *n* = 6; KO, *n* = 4) by the UCSD Animal Care Program Diagnostic Laboratory.

#### Glucose tolerance curve.

Animals were assigned into either a sham (WT, *n* = 5; KO, *n* = 4) or glucose tolerance group (WT, *n* = 8; KO, *n* = 8). Experimental animals were injected with 0.3 g/ml glucose in PBS to achieve a dosage of 2 g/kg. Sham animals were injected with an equivalent amount of PBS. Blood was drawn from the tail at time intervals of 0, 15, 30, 60, and 120 min. Samples were then centrifuged to isolate plasma. Plasma blood glucose was quantified using the Infinity Glucose Kit (Sigma).

#### Calcium uptake measurements.

Intact individual muscle fibers (WT, *n* = 6; KO, *n* = 4) were mechanically dissected from the flexor brevis muscle and loaded with fura-2. Fibers were then stimulated while force generation and Ca^2+^ release were monitored.

#### Four-day endurance test

Endurance was tested by running 24 animals (WT, *n* = 10; KO, *n* = 14) on the Omnipacer Treadmill or the enclosed-chamber modular treadmill using the same exhaustion protocol described above. Mice ran according to this protocol every day for 4 d with a minimum of 22 h of rest between trials. Following the fourth trial, mice were given 24 h of rest and then euthanized. Tissue was harvested and stained using hematoxylin-eosin (as described above) and α-PCNA (Pharmingen, San Diego, California, United States) combined with a DAB Kit (Vector Labs, Burlingame, California, United States).

#### Statistical analysis

Statistical analyses (unpaired Student's t-test, Mann-Whitney test, ANOVA) were carried out using StatView software (SAS Institute, Cary, North Carolina, United States) or Prism software (GraphPad Software, San Diego, California, United States).

## Abbreviations

B-HAD: beta-hydroxyacyl CoA dehydrogenase
CS: citrate synthase
GLUT4: glucose transporter 4
HIF-1α: hypoxia-inducible factor 1α
HIF-1α KO: skeletal-muscle HIF-1α knockout mouse
HMP: hexose monophosphate
LDH-A: lactate dehydrogenase-A
MCK: muscle creatine kinase
PAS: periodic acid–Schiff
PCNA: proliferating cellular nuclear antigen
PCr: phosphocreatine
PFKD: phosphofructokinase disease
PFK-M: muscle-specific form of phosphofructokinase
PGK: phosphoglycerate kinase
PK: pyruvate kinase
RER: respiratory exchange ratio
VEGF: vascular endothelial growth factor
WT: wild-type
